# Case report: Canine distemper virus infection as a cause of central nervous system disease in a Eurasian lynx (*Lynx lynx*)

**DOI:** 10.3389/fvets.2023.1251018

**Published:** 2023-08-14

**Authors:** Mara Sophie Lombardo, Monica Mirolo, Florian Brandes, Albert D. M. E. Osterhaus, Karolin Schütte, Martin Ludlow, Michael Barkhoff, Wolfgang Baumgärtner, Christina Puff

**Affiliations:** ^1^Department of Pathology, University of Veterinary Medicine, Hannover, Germany; ^2^Research Center for Emerging Infections and Zoonoses, University of Veterinary Medicine, Hannover, Germany; ^3^Wildlife Rescue and Conservation Center Sachsenhagen, Sachsenhagen, Germany; ^4^Veterinary Clinic Nienburg, Nienburg, Germany

**Keywords:** computer tomography, encephalitis, endangered species, viral infection, wildlife

## Abstract

The Eurasian lynx (*Lynx lynx*) represents an endangered species with only small populations remaining in Central Europe. Knowledge about the threat posed by potential infectious agents to these animals is crucial for informing ongoing protection measures. Canine distemper virus (CDV) is known to have a wide host range with infection reported in many mammalian species including several lynx species (*Lynx pardinus, Lynx canadensis, Lynx rufus*), but is an extremely rare finding in the Eurasian lynx. The present report describes a case of a Eurasian lynx showing central nervous signs, including apathy and ataxia. A CT scan revealed multiple hypodense areas in different localizations within the brain as well as enlarged liquid filled areas, leading to the suspicion of a degenerative process. Due to clinical deterioration, the animal was euthanized and submitted for macroscopical and histological investigations. Histological investigations revealed multifocal demyelinations in the cerebellum, brain stem and cervical spinal cord as well as a multifocal, perivascular, lymphohistiocytic meningoencephalitis. A CDV infection was confirmed by immunohistochemistry and RT-PCR analyses. This CDV infection of a Eurasian lynx resembles a classical chronic manifestation of distemper in dogs and highlights the threat posed by canine distemper to this species.

## 1. Introduction

The Eurasian lynx (*Lynx lynx*) was extinct in Central Europe prior to the re-establishment of viable populations during reintroduction and protection programs (1). A low reproduction rate and a requirement for large geographical territories are major challenges for the preservation of this species (2). Therefore Central European lynx populations are small and quite isolated, necessitating additional protection measures (1). Knowledge about potential infectious agents is crucial for further preservation of these rare animals, especially regarding bacterial or viral pathogens with a wide host range and the consequent risk of interspecies transmission.

Canine distemper virus (CDV, Canine morbillivirus) is a single-stranded, negative sense RNA virus belonging to the genus *Morbillivirus* in the family *Paramyxoviridae*, and is closely related to human measles virus. The primary target species of CDV infection include many members of the order Carnivora such as dogs (*Canis familiaris*) and wild carnivore species such as the European pine marten (*Martes martes*) and red foxes (*Vulpes vulpes*) (3, 4). The virus can also infect selected non-carnivore species with different degrees and variations of clinical manifestation (4, 5). Some studies have suggested that one or more mutations in the H protein are crucial with respect to infection of new host species (6, 7). The H protein shows a genomic diversity by which it can be categorized into 20 clades, which are mostly related to the geographical area in which they were discovered, with the Arctic, Europe-1 and European wildlife clades all circulating in Central Europe.

In dogs, a CDV infection often manifests initially as a biphasic fever, which might be paralleled or followed by a wide variety of clinical signs (8). The first fever peak is accompanied by infection of lymphoid tissues resulting in a lymphoid depletion during the acute phase of the disease. The second peak occurs in connection with a second viremia and can lead to infection of epithelia in numerous parenchymal organs and tissues. Depending on the involved tissues and organs, respiratory, gastrointestinal, dermal and nervous forms of canine distemper can be observed (3, 8). The latter often manifests in dogs as a characteristic demyelinating leukoencephalitis as a result of chronic CDV infection (9).

In felids, the range of clinical signs after a CDV infection is very wide with experimentally infected domestic cats showing neither symptoms nor virus shedding (10). In contrast, outbreaks with mass mortalities are described in wild large felid populations [Serengeti National Park (11), Ngorongoro Crater (12)]. However, the role of co-infections in the Ngorongoro outbreak is still under debate (12).

CDV infections in different lynx species have been previously described, including the Canadian lynx (*Lynx canadensis*) and the bobcat (*Lynx rufus*) in Canada (13), as well as the Iberian lynx [*Lynx pardinus;* (14, 15)]. Central nervous signs caused by an encephalitis could be confirmed in the cases reported by Daoust et al. in Canada (13). However, to the authors’ knowledge, there is only one previous report of CDV infection in the Eurasian lynx (16), in which the animal showed similar clinical signs as in the present case. Since this animal was identified in Switzerland, the present report describes the first confirmed case of a Eurasian lynx with a CDV infection in Germany.

## 2. Case description

**Figure 1:**
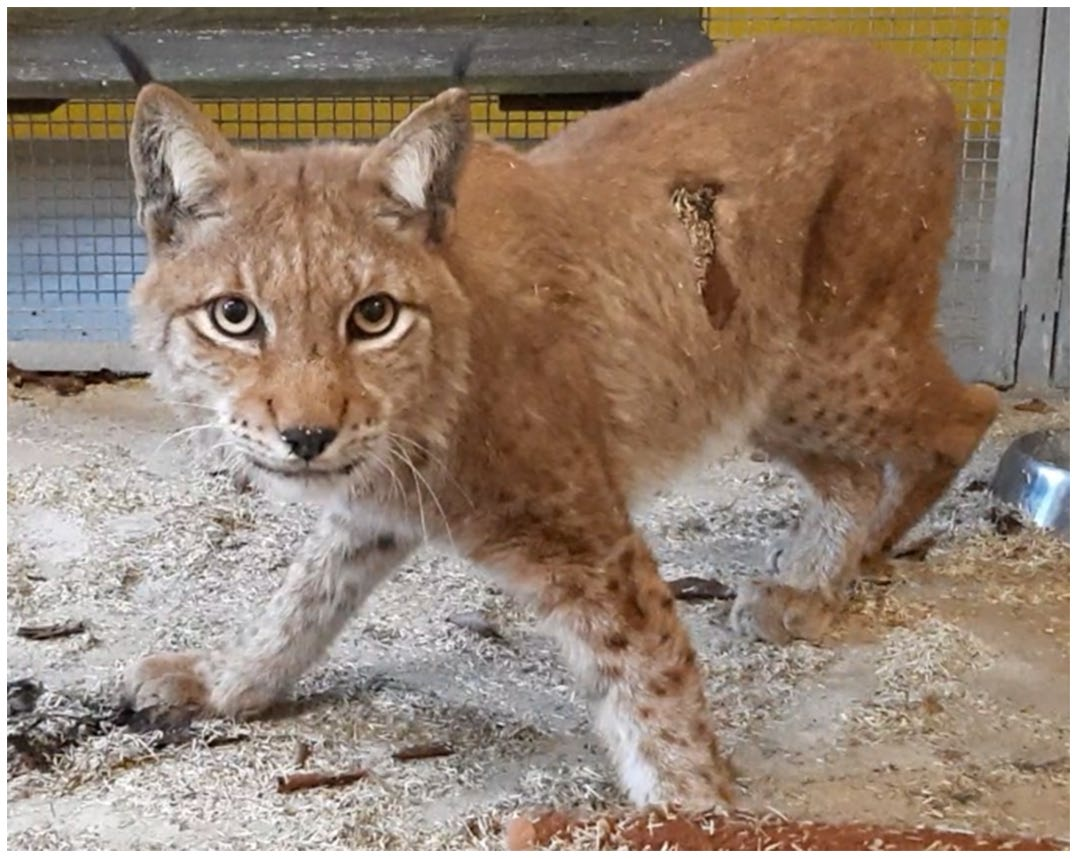
Lynx showing a broad based stand of the front limbs and crossed hind limbs due to ataxia.

**Figure 2:**
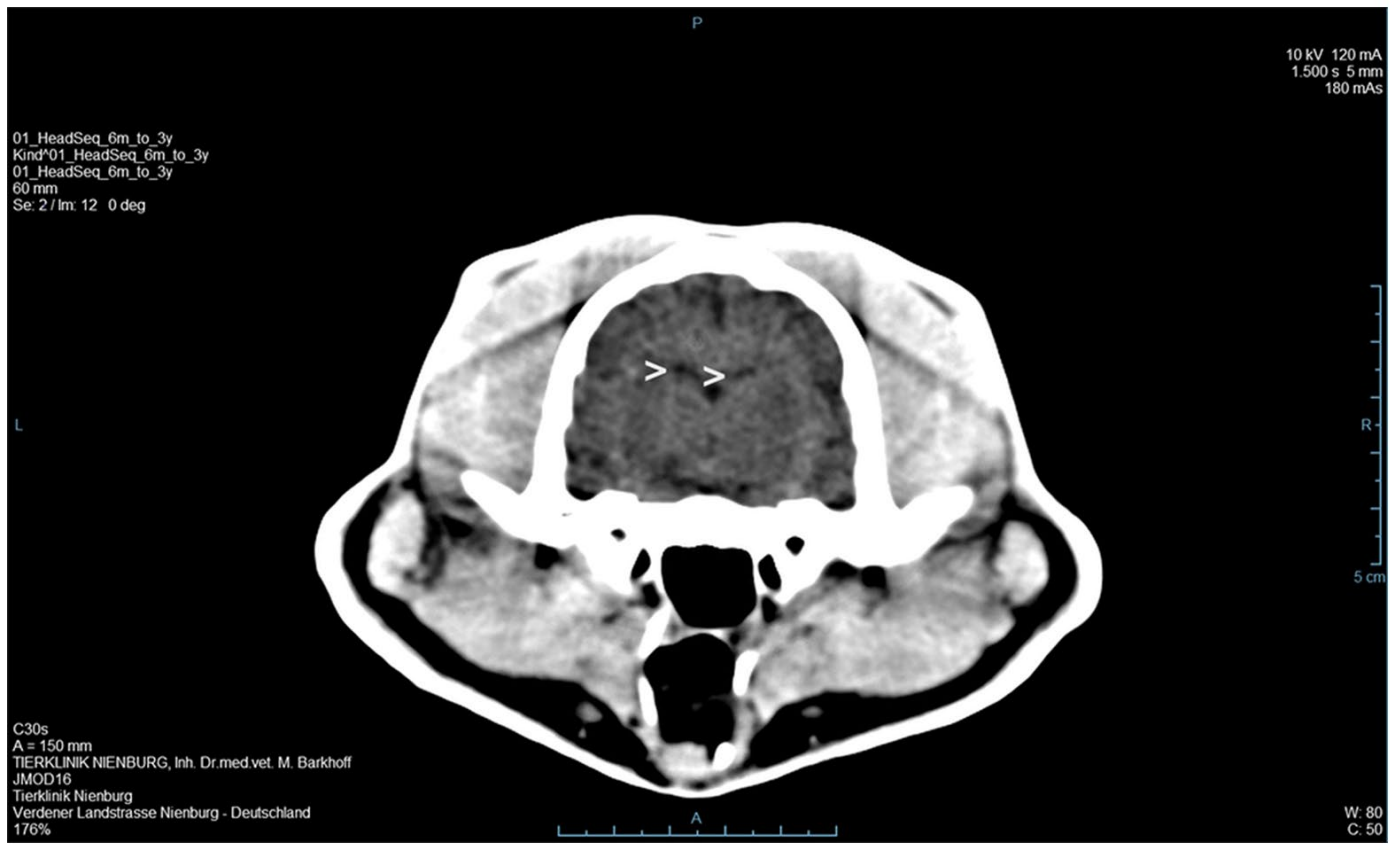
A computer tomographic (CT) scan of the brain revealed a dorsally broadened liquid-filled subarachnoid space as well as multifocal hypodense areas within the parenchyma. Additionally, a decreased ventricular diameter was suspected (**>**).

**Figure 3:**
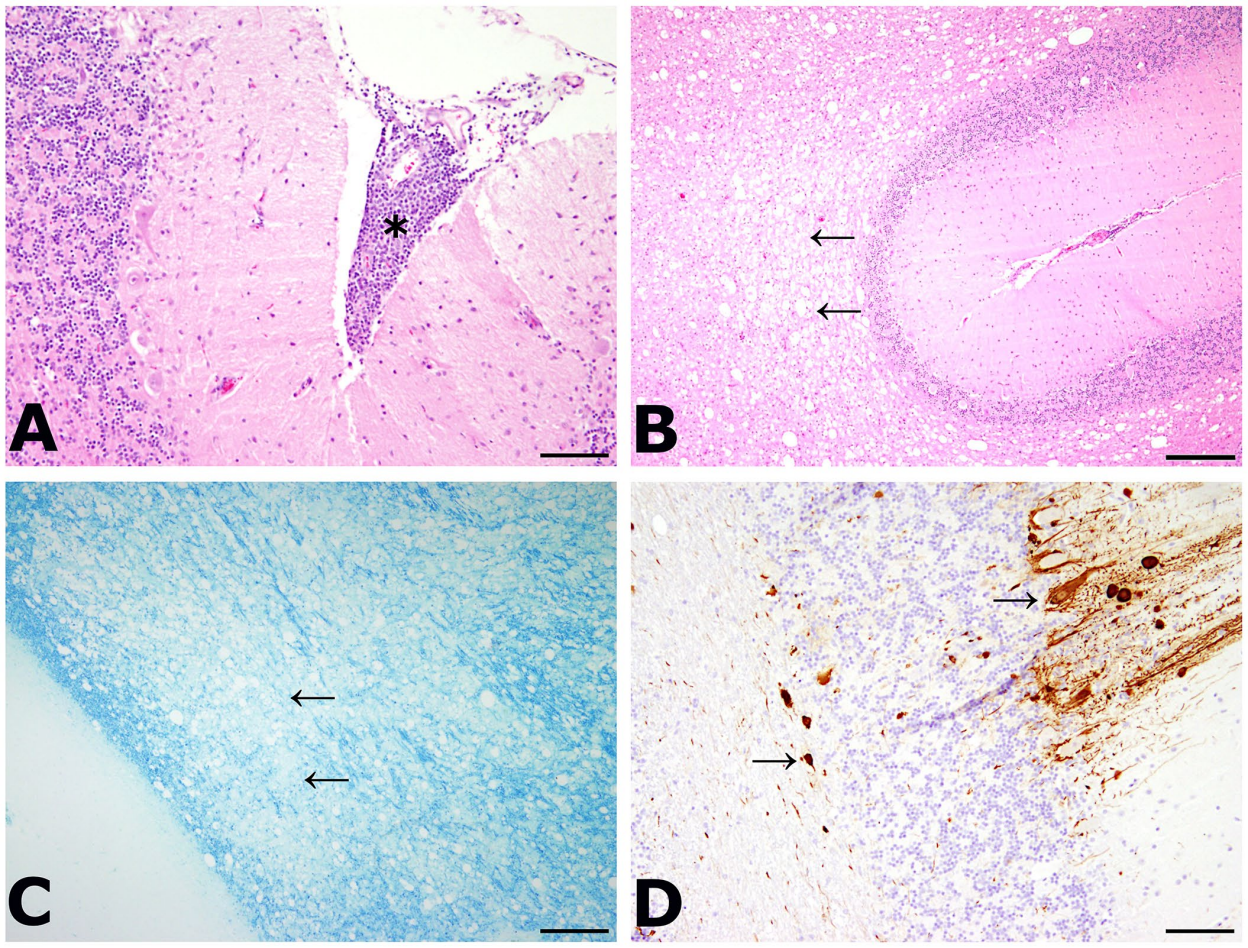
**(A)** Meningoencephalitis in the cerebellum. Inflammatory cells were mainly composed of lymphocytes and macrophages (asterisk). HE, bar: 200 μm. **(B)** Dilation of myelin sheaths in the cerebellum. Multiple vacuoles (arrows) were visible within the white matter of the cerebellum. HE, bar: 500 μm. **(C)** Demyelination of the cerebellum was confirmed by luxol fast blue—cresyl violet staining (LFB/CV). A large area of tissue lacked the blue staining of myelin sheaths (arrows). LFB/CV, bar: 500 μm. **(D)** Detection of viral antigen within the cerebellum. Multiple cells showed an immunolabeling with the anti-CDV-nucleoprotein antibody within the gray and white matter (arrows). CDV nucleoprotein immunohistochemistry (Antibody: D110, Prof. Dr. Zurbriggen, University of Bern), bar: 200 μm.

A 3 year old, male, free ranging Eurasian lynx (*Lynx lynx*) was found in an urban area in Hesse (Taunus) lacking escape reflexes, even when approached by humans. The animal was brought to a wildlife rescue and conservation center for observation. Clinically, the lynx was atactic (see Supplementary Videos 1, 2), with a broad based stand of the front limbs and crossed hind limbs (Figure 1). Furthermore, he showed apathy and a flea infestation. An X-ray investigation as well as a blood examination displayed no further abnormalities. In addition, the animal was tested for different infectious agents. ELISAs detecting feline leukemia virus (FeLV) antigen as well as feline immunodeficiency virus (FIV) and feline coronavirus (FCoV) antibodies were negative. IgG-antibodies against *Toxoplasma gondii* could be detected by an indirect immunofluorescence test (Titer: >1:1,024). The lynx was brought to an animal clinic for sampling of cerebrospinal fluid and a computer tomographic (CT) scan. The CT scan revealed degenerative changes in multiple localizations of the brain such as a dorsally broadened liquid-filled subarachnoid space as well as multifocal hypodense areas within the parenchyma. Furthermore, a generalized edema leading to a decreased diameter of the cerebral ventricles was suspected (Figure 2). In addition, an infection with *Pantoea agglomerans* was detected via microbiological cultivation of cerebrospinal fluid followed by 7 days of treatment with doxycycline. However, no clinical improvement was obtained. Due to worsening of clinical signs, the lynx was euthanized and a necropsy including histology of representative organ samples was performed. For histological investigation, tissue samples were fixed in neutral-buffered formalin (10%) and embedded in paraffin. Sections of 2–3 μm were cut and stained with hematoxylin–eosin (HE) and representative samples of the brain and the spinal cord were additionally stained with luxol fast blue—cresyl violet (LFB/CV). At necropsy no significant morphological findings were observed. Histologically, a multifocal, perivascular, lymphohistiocytic meningoencephalitis was present in cerebrum, cerebellum and brain stem (Figure 3A). In addition, a severe, multifocal dilation of myelin sheaths with multifocal spheroids and myelinophages as well as a mild, multifocal microgliosis was noticed in cerebellum and brain stem (Figure 3B). Similarly, the spinal cord multifocally displayed dilated myelin sheaths with occasional myelinophages. The demyelination was confirmed by LFB/CV staining (Figure 3C). Furthermore, the pulmonary lymph node showed a mild follicular hyperplasia, the mesenteric lymph node displayed a sinus histiocytosis and a mild lymphoid depletion of the tonsils was present. Moreover, a mild, focal, lymphohistiocytic myocarditis was noticed. Apart from agonal changes, no significant findings were observed in the lungs. An immunohistochemical investigation of affected brain regions for CDV nucleoprotein antigen was performed (Antibody: D110, Prof. Dr. Zurbriggen, University of Bern, monoclonal, mouse, 1:1,000 diluted) using the avidin-biotin-complex method with 3,3′-diaminobenzidine (DAB) as a chromogen as described elsewhere (17, 18). CDV-nucleoprotein was detected intralesionally in the gray and white matter of cerebrum and cerebellum (Figure 3D). There was no CDV nucleoprotein detectable within the spinal cord. Additionally, within myocardium, liver, lungs, representative lymph nodes, spleen and intestine CDV nucleoprotein was not detected. The CDV infection of the central nervous system was confirmed on a molecular level via reverse transcriptase polymerase chain reaction (RT-PCR) and Sanger sequencing using hemagglutinin (H) specific primers as previously described (19) using frozen CNS tissue. Analysis of the resulting H gene sequence (GenBank accession no. OR161408) showed only one non-synonymous amino acid substitution (I55T) in comparison to three Europe-1 strains which were previously identified in a fox (GenBank accession no. OL795426) and two raccoons (GenBank accession nos. MN267060 and MN267062) in Germany in 2015. *Toxoplasma gondii* (Antibody: Biocyc GmbH & Co. KG, Potsdam, Germany, polyclonal, rabbit, 1:75 diluted) and feline leukemia virus (Antibody: C11D8; Custom Monoclonals International Corp., Sacramento, United States, monoclonal, mouse, 1:200 diluted) antigens were not detected in the central nervous system using immunohistochemistry.

## 3. Discussion

The lynx displayed a demyelinating meningoencephalitis caused by an infection with canine distemper virus. The histological lesions in the central nervous system are similar to the typical findings observed in the chronic phase of nervous distemper in dogs (3). Daoust et al. made similar observations regarding CDV manifestations in six Canadian lynx and one bobcat in Canada. Five of those animals were further investigated with RT-PCR and nucleotide sequencing and in all but one case, a CDV infection was confirmed. Affected animals of the Canadian study showed varying degrees of microgliosis and perivascular cuffing, as was also observed in the present case. While demyelination was a very prominent finding in the current study, only a subset of the lynx investigated by Daoust et al. exhibited this pathological change. In contrast to our findings, Daoust et al. also observed neuronal necrosis in most lynx (13). Regarding diagnostic imaging, the findings of the CT scan in the present case also show similarities to changes observed with magnetic resonance imaging (MRI) in CDV infected dogs. In the literature, a correlation of MRI and histological findings of demyelination in the brainstem and cerebellum was observed and MRI has been shown to be a sensitive tool for the diagnosis of demyelination (20), which might also be the case for CT scans considering the results of the present case.

In other outbreaks of CDV in wild large felids, the role of co-infections, which led to an immunosuppression is still discussed (12). In the present case, there were no morphological indications for such a co-infection. The detection of *Pantoea agglomerans* in the cerebrospinal fluid most likely represents a contamination, since these bacteria are found ubiquitously and can often be isolated from plant surfaces or feces (21). Furthermore, an antibiotic treatment did not improve clinical signs rendering an etiological relevance of the isolated bacteria less likely. Although the animal of the present case showed antibodies against *Toxoplasma gondii,* there were no signs for an ongoing infection with this agent and the immunohistochemical investigation was negative. However, we found an infection with a Europe-1 strain of CDV of the lynx. The red fox, representing the most abundant canid wildlife population in Europe, as well as European domestic dogs have been reported to be more susceptible to Europe-1 strains than to European wildlife strains (7). Regardless of the strain origin, raccoons, martens or foxes are quite often infected with CDV and thus represent a common reservoir (3) and a potential source of infection in the present case. There were no mutations identified within the receptor binding sites of the H protein, indicative of alterations to usage of the cellular receptors CD150 or Nectin 4. In Iberian lynx, a study suggested, that bacterial and protozoal infections occur more frequently than viral infections due to the free ranging, solitary lifestyle of lynx (22). Due to this solitary lifestyle and the rare reports of Eurasian lynx infected with CDV, the animal in the current case was most likely infected via virus transmission from another species such as a raccoon, martens or domestic dogs, than another CDV-infected lynx. Additionally, in other CDV outbreaks in large wild felids, virus strains were found to be closely related to strains identified in domestic dogs present in the same geographical region (11).

## 4. Conclusion

The Eurasian lynx is a rare species, which needs to be protected. Knowledge about potential infectious agents is therefore crucially important. Furthermore, canine distemper should be included to the list of differentials in case of central nervous signs in lynx.

## Data availability statement

The datasets presented in this study can be found in online repositories. The names of the repository/repositories and accession number(s) can be found at: https://www.ncbi.nlm.nih.gov/genbank/, OR161408.

## Ethics statement

Ethical review and approval was not required for the animal study because the presented case was initially submitted for routine diagnostic purposes (necropsy for determination of the cause of disease).

## Author contributions

FB, KS, MB, MLu, MM, and MLo: sample collection and data generation. CP, FB, KS, MB, MLu, and MLo: methodology, data analysis and interpretation. CP, MLu, and WB: supervision. MLo: visualization. AO, MLu, and WB: resources. MLo and CP: writing—original draft preparation. AO, CP, FB, KS, MB, MLu, MM, MLo, and WB: writing—review and editing. All authors contributed to the article and approved the submitted version.

## Funding

This research was in part funded by the Deutsche Forschungsgemeinschaft (DFG, German Research Foundation)-398066876/GRK 2485/1-VIPER-GRK. This Open Access publication was funded by the Deutsche Forschungsgemeinschaft (DFG, German Research Foundation)—491094227 “Open Access Publication Funding” and the University of Veterinary Medicine Hannover, Foundation.

## Conflict of interest

The authors declare that the research was conducted in the absence of any commercial or financial relationships that could be construed as a potential conflict of interest.

## Publisher’s note

All claims expressed in this article are solely those of the authors and do not necessarily represent those of their affiliated organizations, or those of the publisher, the editors and the reviewers. Any product that may be evaluated in this article, or claim that may be made by its manufacturer, is not guaranteed or endorsed by the publisher.
